# Gender Differentiation of Relationships Between Manifestations of Indirect Self-Destructiveness and Dimensions of Emotional Intelligence

**DOI:** 10.1007/s11126-015-9402-2

**Published:** 2015-11-20

**Authors:** Konstantinos Tsirigotis, Joanna Łuczak

**Affiliations:** Department of Psychology, The Jan Kochanowski University in Kielce, Piotrków Trybunalski Branch, Słowackiego 114/118 str., 97-300 Piotrków Trybunalski, Poland

**Keywords:** Manifestations of indirect self-destructiveness, Dimensions of emotional intelligence, Gender

## Abstract

The aim of this study has been to explore the gender differentiation of relationships between individual manifestations of indirect self-destructiveness and particular dimensions of emotional intelligence. A population of 260 individuals (130 women and 130 men) aged 20-30 (mean age of 24.5) was studied by using the Polish version of the Chronic Self-Destructiveness Scale (CS-DS) and INTE, i.e. the Polish version of the Assessing Emotions Scale (AES). Manifestations of indirect self-destructiveness showed significant correlations with INTE variables, and those correlations were mainly negative. Relationships between specific dimensions of emotional intelligence and specific manifestations of self-destructiveness differed between women and men. One of the most important differences was the relationship between transgression and ability to recognize emotions. The knowledge of the differentiation of the above relationships may allow to orient prophylactic and therapeutic actions, and adjust them to the specific gender.

## Introduction

The different experiencing and understanding of the world by women and men is quite a well scientifically substantiated psychological and sociological fact. A similar phenomenon is observed for psychological functioning as well as experienced and manifested psychopathology [cf. 1–8].

Gender differences in the psychological functioning and psychopathology are also noticeable in the spheres of emotional intelligence and indirect self-destructiveness. Women show higher emotional intelligence than men [9–13], whereas indirect self-destructiveness is more intense in men or even associated with the psychological dimension of masculinity, irrespective of the sex [7, 14].

Behaviours causing harm to the subject are called self-destructive behaviours. Two basic forms of self-destructive behaviours can be distinguished: direct (open, acute) and indirect (latent, chronic) [15–17].

Kelley describes chronic self-destructiveness as a generalised tendency to undertake behaviours increasing the probability of negative and decreasing the probability of positive consequences for the subject [15]. For the purposes of this study, it was assumed that indirect/chronic self-destructiveness comprises behaviours whose probable negative effect is intermediated by additional factors, while the relationship between a behaviour and harm is perceived as probable. Indirect self-destructiveness understood in such a way includes both taking and abandoning specific actions; it concerns getting into hazardous and increased-risk situations (active form) or neglecting one’s safety or health (passive form). Moreover, indirect self-destructiveness is a form of self-destruction characterised by an increased temporal distance between an action and its effect [16, 17]. There are, in general, several categories of indirectly self-destructive behaviours: transgression and risk, poor health maintenance, personal and social neglects, lack of planfulness, and helplessness and passiveness when facing problems/difficulties. Transgression and risk are behaviours violating social norms, such as school rules or principles of community life, as well as risky behaviours undertaken for a momentary pleasure, e.g. driving with bravado connected with a desire to impress others, feel appreciated, better or noticed, or gambling. That category also comprises succumbing to temptations, impulsiveness, and seeking risky excitation. Poor health maintenance encompasses behaviours harmful to one’s health, such as excessive eating or drinking, missing medical appointments or ignoring physicians’ instructions. Personal and social neglects include, for instance, neglecting one’s duties or matters (personally and interpersonally) important to the subject. Lack of planfulness consists in acting mainly on the spur of the moment with nothing in view. Helplessness and passiveness mean giving up an action or not taking that in circumstances where such an action might stop suffering or prevent a danger [15–17].

Indirect self-destructiveness is a form of harming oneself that distinctly differs from direct self-destructiveness or self-aggression. The essence of indirect self-destructiveness is its trans-situational nature and the co-occurrence of various forms of behaviours that lead to adverse consequences. It is not a coincidence that indirect-self destructiveness is referred to as “slow” or “lingering” suicide.

In turn, emotional intelligence is a psychological phenomenon (trait, ability) beneficial to the human being. The second half of the 20th century saw the occurrence of hypotheses proposing that emotions may positively affect mental processes and psychological functioning in general [cf. 18].

According to Salovay and Mayer’s model, emotional intelligence is a set of abilities and a subset of social intelligence that includes the following three categories of adaptive abilities: appraisal and expression of emotions, regulation of emotions and utilisation of emotions in problem solving. The first category consists of components of appraisal and expression of one’s own emotions and appraisal of emotions of others. The component of appraisal and expression of one’s own emotions is further divided into two subcomponents, i.e. verbal and non-verbal, while the component of appraisal of emotions of others is divided into subcomponents of non-verbal perception and empathy. The second category of emotional intelligence—regulation—includes components of regulation of emotions in self and regulation of emotions in others. The third category—utilisation of emotions—incorporates components of flexible planning, creative thinking, redirected attention and motivation. Even though emotions are at the core of the model, it also includes social and cognitive functions connected with the expression, regulation and utilization of emotions [11, 18]. Mayer et al. [2004] further developed that model but, in the opinion of many authors, fundamental aspects of emotional intelligence proposed in the latest model are similar to those contained in the 1990 one [cf. 19].

Consequently, individuals who have developed abilities connected with emotional intelligence understand and express their own emotions, recognize emotions of others, regulate affect, and utilise moods and emotions to motivate adaptive behaviours [18]. Authors wonder whether it is not yet another definition of a healthy, self-actualising individual.

Research has shown that individuals who are primarily motivated by current emotional factors are more likely than those motivated by more distant cognitive considerations to engage in acts that are ultimately self-destructive. Generally, those individuals who are more responsive to immediate emotional factors than to more distant rational projections of consequences are likely to engage in maladaptive acts. Though the specific acts in question vary widely, the unifying characteristic seems to be response to affect rather than to cognitions. Every behaviour appears to represent the tendency to seek immediate pleasure or avoid immediate discomfort, regardless of the long-term consequences of doing so [15].

While emotional intelligence may have a favourable influence on the life and psychological and social functioning of the individual, indirect self-destructiveness exerts a rather negative influence. World literature offers almost no studies into relationships between indirect self-destructiveness and emotional intelligence. As a result of recently carried out research, it was found that indirect self-destructiveness as a generalised behavioural tendency negatively correlates with emotional intelligence [20]. Thus, the relationship between those two aspects of psychological functioning is inversely proportional: the higher the indirect self-destructiveness the lower the emotional intelligence and vice versa: the lower the indirect self-destructiveness the higher the emotional intelligence. In other words, these variables negatively correlate with each other: emotional intelligence protects against indirect self-destructiveness, while indirect self-destructiveness disturbs or even damages emotional intelligence [20, 21].

For research and practical reasons, it is interesting to examine relationships between manifestations of indirect self-destructiveness and dimensions of emotional intelligence separately in the population of women and in the population of men.

The aim of this study has been to explore gender differentiation of relationships between individual manifestations of indirect self-destructiveness and particular dimensions of emotional intelligence.

## Methods

The study is part of two more extensive research projects (on indirect self-destructiveness and on emotional intelligence) and thus the applied methodology or some other parts of the studies may be similar.

### Participants

A population of 260 individuals (130 women and 130 men) aged 20–30 (mean age of 24.5) was studied by using the Polish version of the Chronic Self-Destructiveness Scale (CS-DS) by Kelley et al. [15] in its adaptation by Suchańska [16] and the Polish version of the Assessing Emotions Scale (AES) by Schutte et al. [11] in its adaptation by Jaworowska et al. [22]. The study group was formed on the basis of the random selection from the general population (of healthy subjects); participation in the study was voluntary and anonymous, and consistent with the principles of the Declaration of Helsinki.

## Materials

In order to examine indirect (chronic) self-destructiveness as a generalized tendency, Kelley created a research tool comprising several categories of indirectly self-destructive behaviour; the ultimate version was made up of a Likert-type internally consistent set of 52 items with the total obtained score indicating the intensity of indirect self-destructiveness. The research tool encompasses the following categories: Transgression and Risk (A1), Poor Health Maintenance (A2), Personal and Social Neglects (A3), Lack of Planfulness (A4), and Helplessness and Passiveness in the face of problems/difficulties (A5), the scores for which sum up to one global score for indirect self-destructiveness. Both the original scale and its Polish adaptation are characterised by high reliability and validity [15, 16].

Schutte et al. [11] created a tool to examine emotional intelligence. Since then, the questionnaire has been used in many studies, although under different names [9, 13, 23–25]. This study applies the Emotional Intelligence Questionnaire INTE, i.e. the Polish version of the AES, as adapted by Jaworowska et al. [22]. The questionnaire is composed of 33 items on which the subject may take a position by choosing one of the five possible answers (the Likert-type scale). Along with the general emotional intelligence score, the scale enables to receive scores for two factors: Factor I is ability to utilise emotions in order to support thinking and actions, while Factor II is ability to recognise emotions. Both the original and Polish versions are characterised by high reliability and validity [11, 22].

## Statistical Analysis

The statistical analysis of received scores applied descriptive methods and statistical inference methods. In order to describe the mean value for quantitative traits, the arithmetic mean (M) was calculated, while the standard deviation (SD) was assumed to be the dispersion measure. The conformity of distributions of quantitative traits with the normal distribution was assessed using the Shapiro–Wilk test. Due to the lack of conformity of distributions of dependent variables with the normal distribution, the statistical processing of received results used non-parametric statistics; in order to examine the relationship between the studied variables, Kendall’s “tau” (τ) correlation coefficient was used. For all the analyses, the maximum acceptable type I error was assumed at α = 0.05. Asymptotic two-sided probability test p was calculated and *p* ≤ 0.05 was considered to indicate statistical significance. The statistical analyses were performed by means of the *Statistica PL 12.5 for Windows* [26] statistical package.

## Results

As a result of a recently carried out research project, statistically significant negative correlations were found between indirect self-destructiveness as a generalised behavioural tendency and the dimensions of emotional intelligence [20]. Moreover, many statistically significant correlations were revealed between specific indirect self-destructiveness categories and particular dimensions of emotional intelligence, which were negative, too [21].

**Fig. 1:**
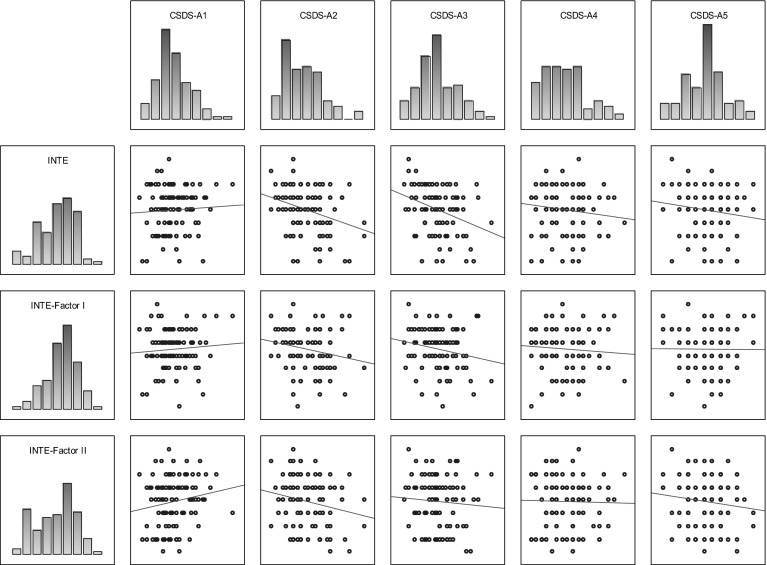
Scatterplot matrix of variables scores (INTE, CS-DS) in the group of women

Table 1 shows correlation coefficients (Kendall’s tau) between the studied variables using the CS-DS and INTE in the group of women; Fig. 1 shows the scatterplot matrices of those scores.

**Table 1:** Correlation coefficients between variables measured by CS-DS and INTE in the group of women

VARIABLE	CS-DS-A1	CS-DS-A2	CS-DS-A3	CS-DS-A4	CS-DS-A5
INTE	−0.002 ns.	−0.298 *p*: 0.000003	−0.321 *p*: 0.0000001	−0.166 *p*: 0.005	−0.151 *p*: 0.01
INTE-Factor I	−0.01 ns.	−0.257 *p*: 0.00005	−0.193 *p*: 0.02	−0.141 *p*: 0.002	−0.058 ns.
INTE-Factor II	+0.184 *p*: 0.007	−0.244 *p*: 0.00004	−0.059 ns.	−0.030 ns.	−0.148 *p*: 0.03

*CS-DS* polish version of the “Chronic Self-Destructiveness Scale”, *CS-DS-A1* transgression and risk, *CS-DS-A2* poor health maintenance, *CS-DS-A3* social and personal neglects, *CS-DS-A4* lack of planfulness; *CS-DS-A5* helplessness and passiveness in the face of problems, *INTE* polish version of the “Assessing Emotions Scale”, *INTE-Factor I* ability to utilise emotions, *INTE-Factor II* ability to recognise emotions

The group of women displayed the same correlations as those in the whole population; in the group of women the distribution of correlation coefficients was similar to the distribution for the whole studied population, i.e. similar correlations occurred [cf. 21]. The only exception was the correlation between INTE Factor II and CS-DS scale A1 (Transgression and Risk): while that coefficient was statistically non-significant for the whole population, in the group of women it was significant and bore the same sign, i.e. was positive (although all the other correlation coefficients were negative). It was the sole statistically significant positive correlation both in the whole population and in the group of women. At the same time, it is an answer to the question asked in a previous work [21], namely: why did the only positive correlation coefficient occur between those two variables? It was the distribution of scores in the group of women that caused that to happen. No correlation coefficients were found common only to the group of women and the group of men: if a given correlation coefficient was common to the women and men, it also occurred in the whole population. Based on the above data, a hypothesis can be put forward that it was the women that affected the relationship between the INTE and CS-DS variables in the whole population. Ten statistically significant correlation coefficients were found between scores for the variables studied by means of specific CS-DS and INTE scales and indices.

The INTE (total score) correlated negatively with the following four CS-DS scales: A2 (Poor Health Maintenance), A3 (Personal and Social Neglects), A4 (Lack of Planfulness), and A5 (Helplessness, Passiveness in the face of problems/difficulties).

INTE Factor I (ability to utilize emotions in order to support thinking and actions) correlated negatively with the following three CS-DS scales: A2 (Poor Health Maintenance), A3 (Personal and Social Neglects), and A4 (Lack of Planfulness).

INTE Factor II (ability to recognise emotions) correlated negatively with the following two CS-DS scales: A2 (Poor Health Maintenance) and A5 (Helplessness, Passiveness in the face of problems/difficulties). However, the same Factor II correlated positively with CS-DS scale A1 (Transgression and Risk); as already mentioned, it was the only statistically significant positive correlation coefficient in the whole population and in both the groups.

The highest correlation coefficient occurred between the INTE (total score) and CS-DS A3 scale (Personal and Social Neglects) being −0.321 (p: 0.0000001). It was only scale CS-DS A2 (Poor Health Maintenance) that correlated with all the INTE variables.

**Fig. 2:**
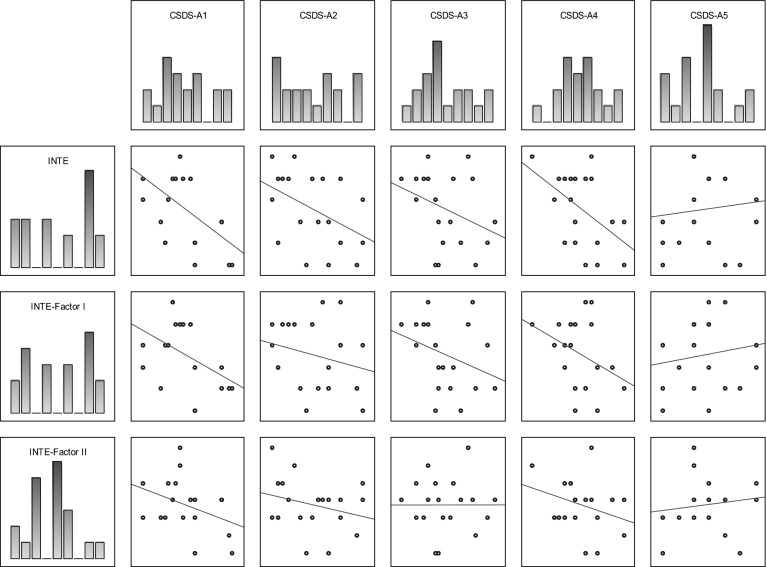
Scatterplot matrix of variables scores (INTE, CS-DS) in the group of men

Table 2 shows correlation coefficients (Kendall’s tau) between the studied variables using the CS-DS and INTE in the group of men; Fig. 2 shows the scatterplot matrices of those scores.

**Table 2:** Correlation coefficients between variables measured by CS-DS and INTE in the group of men

VARIABLE	CS-DS-A1	CS-DS-A2	CS-DS-A3	CS-DS-A4	CS-DS-A5
INTE	−0.544 *p* < 0.0000000	−0.458 *p*: 0.004	−0.420 *p*: 0.008	−0.497 *p*: 0.002	+0.08 ns.
INTE-Factor I	−0.454 *p*: 0.004	−0.158 ns.	−0.385 *p*: 0.01	−0.402 *p*: 0.01	+0.157 ns.
INTE-Factor II	−0.486 *p*: 0.002	−0.367 *p*: 0.02	−0.087 ns.	−0.336 *p*: 0.03	+0.126 ns.

*CS-DS* polish version of the “Chronic Self-Destructiveness Scale”, *CS-DS-A1* transgression and risk, *CS-DS-A2* poor health maintenance, *CS-DS-A3* social and personal neglects, *CS-DS-A4* lack of planfulness, *CS-DS-A5* helplessness and passiveness in the face of problems

In the group of men, slightly different variables statistically significantly correlated with one another; the differences concerned mainly the correlations of CS-DS scales A1 (Transgression and Risk) and A5 (Helplessness and Passiveness); although the latter did not reach statistical significance. Furthermore, correlation coefficients were higher in the group of men than in the group of women and in the whole population.

It should be emphasized that all statistically significant correlation coefficients in the group of men, without exception, were negative, i.e. it was different than in the group of women, where one statistically significant positive correlation coefficient was detected (between INTE Factor II and CS-DS scale A1); that coefficient was positive in the whole population too, but without statistical significance [cf. 21]. Ten statistically significant correlation coefficients were found in the group of men, all of which were negative.

The INTE (total score) correlated negatively with four CS-DS scales: A1 (Transgression and Risk), A2 (Poor Health Maintenance), A3 (Personal and Social Neglects), and A4 (Lack of Planfulness).

INTE Factor I (ability to utilise emotions in order to support thinking and actions) correlated negatively with three CS-DS scales: A1 (Transgression and Risk), A3 (Personal and Social Neglects), and A4 (Lack of Planfulness).

INTE Factor II (ability to recognise emotions) also negatively correlated with three CS-DS scales: A1 (Transgression and Risk), A2 (Poor Health Maintenance), and A4 (Lack of Planfulness).

As can be seen, as opposed to the whole population [cf. 21] and the group of women, in the group of men there were no statistically significant correlations between CS-DS scale A5 (Helplessness, Passiveness in the face of problems/difficulties) and the INTE variables.

It should also be noted that, as opposed to the group of women and the whole population (where negative correlations were observed), scale A5 (Helplessness and Passiveness in the face of problems/difficulties) correlated positively with all the INTE variables although the correlations were statistically non-significant.

Moreover, also unlike in the whole population and the group of women, in the group of men scale CS-DS A1 (Transgression and Risk) significantly (and negatively) correlated with all the INTE variables; in turn, as we could observe, in the group of women the only significant correlation of scale CS-DS A1 was with INTE Factor II and it was positive, as opposed to all the other significant correlations.

The highest correlation coefficient in the group of men occurred between the INTE (total score) and CS-DS A1 (Transgression and Risk) at −0.544 (*p* < 0.000000).

The results of these analyses confirm the results of the negative correlations between CS-DS categories and INTE dimensions in both the groups: the higher the scores in the INTE dimensions, the lower the scores in the CS-DS categories and vice versa: the lower the scores in the INTE, the higher the scores in the CS-DS. There were few, although notable, exceptions.

## Discussion

Since literature offers a scarce number of studies into relationships between manifestations of indirect self-destructiveness and dimensions of emotional intelligence, especially into their gender differentiation, it will be difficult to refer to results of other studies.

As already stated, emotional intelligence is a psychological entity (trait, ability) creating favourable conditions for the psychological, social and even physical well-being of the human, whereas the indirect self-destructiveness syndrome is rather harmful to that. Therefore, it can be assumed that emotional intelligence protects against indirect self-destructiveness, while indirect self-destructiveness interferes with, disturbs or even damages both emotional intelligence and human well-being as a whole [20].

In this study, we decided to take a closer look at relationships between specific dimensions or factors of emotional intelligence and specific categories of indirectly self-destructive behaviours for women and men separately.

As already mentioned, almost all correlations (especially in the whole population and in the group of women) carried the minus sign, regardless of the significance level, which can mean that those two types of psychological phenomena (traits) are in opposition to each other. In this part of the study, we are going to deal only with relationships that reached statistical significance. We will start with common relationships, i.e. those that occurred both in the entire population and in each of the groups (of women and men).

### Women and Men

As mentioned above, there were no correlations common only to women and men: all relationships occurring in both the groups occurred also in the whole population.

Poor health maintenance (A2) correlated negatively with emotional intelligence as a whole and ability to recognise emotions. Thus, a conclusion can be drawn that emotional intelligence as a whole, as well as the ability to recognize emotions, protect the individual’s psychophysical health. That is an empirical proof of the fact that there are positive relationships between emotional intelligence, and that of its components, and health in general. Observations in line with the above were also made by other authors [12, 18, 20, 27, cf. 21].

Another category of indirectly self-destructive behaviours, personal and social neglects (A3), was also negatively correlated with emotional intelligence in general, as well as ability to utilize emotions in order to support thinking and actions. Helping others may require sacrifices and emotional toughness [18]. Other authors also report negative correlations between emotional intelligence and deviant social behaviours [28]. Individuals having most serious problems with respecting others, i.e. prisoners (criminals), have low emotional intelligence [11]. Higher emotional intelligence is associated with better psychosocial functioning, including intrapersonal factors (such as higher optimism) and interpersonal factors (such as better interpersonal, social relations) [12]. Furthermore, individuals with high emotional intelligence show more empathy in relations with others, more self-monitoring in social situations, as well as more closeness and warmth in relations with others [13, 29].

Authors of the emotional intelligence concept report ability to predict, prevent and counteract adjustment disorders such as aggression and violence [30]. A dramatic manifestation of intrapersonal and interpersonal dysfunctions is domestic violence. Perpetrators of domestic violence (mostly men) have lower emotional intelligence than the general population; moreover, emotional intelligence deficits are connected with a tendency to use violence in both the group of violence perpetrators and the general population [31]. Even women suffering domestic violence have lower emotional intelligence than women who do not suffer that [32].

Empathy and self-monitoring in social situations [13, 29] may protect against (prevent) disorders of social and personal functioning. Another well-known researcher of emotional intelligence, Bar-On, also states that emotionally intelligent individuals adjust better in their environment, including the social one [33].

Lack of planfulness (A4) also negatively correlated with emotional intelligence as a whole and as ability to utilise emotions in order to support thinking and actions. Individuals who cannot recognise their own emotions are unable to plan their lives in order to find fulfilment; such planning deficits may lead to feeling the lack of meaning of life which affects depressive individuals and even those having suicidal ideations [18]. Assuming that academic achievements result also from ability to plan, it was stated that emotional intelligence is a good predictor of high achievements at university [11].

Ability to utilise emotions may be helpful in planning one’s own actions and predicting their consequences to be of benefit to oneself and others. That way of acting usually results in better adjustment and more effective coping in the environment [18, 27, cf. 21]. As already mentioned, the above relationships occur also in the whole population [cf. 21].

### Women

As mentioned above, women influenced the relationships (associations) between the INTE and CS-DS variables in the whole population: there where the same relationships in the whole group as in the group of women.

Poor health maintenance (A2) negatively correlated with all dimensions or factors of emotional intelligence in the group of women, as opposed to the group of men, where there was no relationship with ability to utilise emotions; that would mean that men, although able to recognise emotions, may have problems with utilising them in the sphere of protecting their health. Thus, a conclusion can be drawn that, in the case of women, emotional intelligence as a whole and its specific dimensions (components), i.e. ability to recognise emotions and ability to utilise emotions in order to support thinking and actions, protect the individual’s psychophysical health. It is an empirical proof of the fact that there are positive relationships between emotional intelligence and its components and health in general. Individuals with developed emotional intelligence abilities recognise emotions (of their own and others) as well as utilise moods and emotions to motivate adaptive behaviours [18, 20]. It is consistent with the statement that higher emotional intelligence is associated with better psychophysiological health [12, 27]. That may work based on a mechanism, e.g. of preventive actions in the case of the so called prodromal asthenia or starting reaction [34] often preceding a medical condition: individuals with higher emotional intelligence may recognise psychological prodromal symptoms of a somatic disease and make attempts at treatment early enough. In turn, in the case of falling ill such a person follows the physician’s instructions and better cooperates with the physician (compliance), on the one hand, thanks to the awareness of one’s own state and consequences of one’s own actions but, on the other hand, also owing to the phenomenon of “emotional exchange” with the healthcare professional.

Some psychosocial factors, such as stronger social support and greater satisfaction with social support in individuals with higher emotional intelligence, may serve as buffers against somatic diseases. Moreover, individuals with higher emotional intelligence can, to a larger extent, act according to principles of health behaviour and show better medical compliance [12].

Therefore, individuals with higher emotional intelligence tend to be in a positive mood and easier elevate that when they sometimes are in a negative one [12, 27, cf. 21]. Moreover, women in general display less poor health maintenance than men [7].

The last category of indirectly self-destructive behaviours, helplessness (A5), negatively correlated with general emotional intelligence and ability to recognise one’s own emotions and emotions of others. Such a result may suggest that emotional intelligence in general, and ability to recognise emotions in particular, protect against the lack of ability to cope with problems and abandoning or refraining from taking remedial measures in difficult situations. The lack of motivation or readiness to take active measures in the face of difficulties or their total abandonment cause further, secondary psychological, health-related and social damage. Emotional intelligence protects against depression and the feeling of hopelessness and helplessness [20, 28]. On the other hand, emotional intelligence is connected with greater optimism and absence of depressive states [13, 20, 29]. The absence of helplessness may be a kind of bridge to psychophysical health. As mentioned earlier, higher optimism and the sense of receiving social support may constitute buffers against a somatic disease [12]. Some authors [35] propose to call emotional intelligence emotional self-efficacy, while self-efficacy is the opposite or even contradiction of self-handicapping, being one of the major components of indirect self-destructiveness, especially of helplessness and passiveness [cf. 20].

### Men

As mentioned above, correlation coefficients in the group of men were higher than in the group of women, which can mean that relationships between indirect self-destructiveness categories and emotional intelligence dimensions were stronger in the group of men than in the group of women, regardless of their direction (sign).

It was only in the group of men that transgression and risk (A1) showed relationships with both the abilities making up emotional intelligence, and those were negative, unlike in the group of women, where A1 correlated positively with ability to recognise emotions. Such a result can become easier to understand if we take into account the fact that transgression and risk are more intense in men [7]. It is also important that it was that category of indirectly self-destructive behaviours that displayed the strongest and negative relationships with emotional intelligence in the group of men. Results of other studies indicated that emotional intelligence is associated with lower impulsiveness [11, 13], which is an important element of indirect self-destructiveness. In turn, the lack of awareness of emotions and inability to manage emotions are key symptoms in impulse control disorders [12, 36]. Therefore, it can be assumed that emotional intelligence, both as ability to recognize emotions and ability to utilise emotions in life, protects men from undertaking risky behaviours that can have even fatal consequences for them.

Lack of planfulness (A4) also negatively correlated with ability to recognise emotions solely in the group of men. Individuals who cannot recognize their own emotions are unable to plan their lives in order to find fulfilment; such planning deficits may lead to feeling the lack of meaning of life which affects depressive individuals and even those having suicidal ideations [18]. Assuming that academic achievements result also from ability to plan, it was stated that emotional intelligence is a good predictor of high achievements at university [11]. Ability to recognise emotions may be helpful in planning one’s own actions and predicting their consequences to be of benefit to oneself and others. That way of acting usually results in better adjustment and more effective coping in the environment [18, 27, cf. 21].

### Opposite Relationships

It is worth noting the only, in the whole population and both the groups, positive correlation between transgression and risk (CS-DS A1) and ability to recognize emotions (INTE Factor II). As mentioned above, such a result impacted on the whole population. When trying to interpret such a result, one should remember that transgression and risk are less intense in women [7]. The evolutionary meaning of that finding, according to some researchers, may indicate a lower propensity of women for undertaking risky behaviours, connected with the necessity to protect offspring and hence more conservative forms of behaviour [cf. 37, 38]. Nevertheless some authors point out certain positive aspects of risk-taking, claiming that risk-takers cope well with stress: propensity for risk-taking may be, among others, a manifestation of special tolerance and efficacy of coping mechanisms in psychological stress conditions [39, 40]. However, taking into account the fact that women show lower propensity for such behaviours, it can be assumed that the relationship concerns mainly the aspect of going beyond barriers or norms rather than the aspect of strictly risky behaviours; perhaps the adaptive meaning of propensity for transgression and risk can be seen here. Following that line of thinking, it can be assumed that, in women, the recognition of emotions occurs in the form of “going beyond oneself”, towards the other person, i.e. “emotional exchange” and empathy, which is better developed in women [cf. 41, 42]. In turn, in men, the recognition of emotions of others may take place by way of an analogy based on introspection.

What seems puzzling is positive, although statistically non-significant, relationships between helplessness (A5) and emotional intelligence as a whole, as well as its specific dimensions, i.e. ability to recognise and ability to utilise emotions; those relationships were reverse to those in the group of women and the whole population. At that point, it should be reminded that helplessness is more intense in men than in women [7]. In order not to go as far as to draw unjustified conclusions (due to the lack of statistical significance of those correlations), a hypothesis can be put forward that emotional intelligence creates favourable conditions for helplessness in men, and mechanisms causing that phenomenon should be subject to further research. It is a well-known fact that men show lower emotional intelligence than women in general [9–13]. Moreover, it is also a well-known fact that alexithymia, which negatively correlates with emotional intelligence [cf. 13], is stronger and more common in men than in women [43]. Quite possibly, men feel helpless in the face and scope of emotional intelligence. As already mentioned, however, the issue should be further researched.

## Conclusions

Relationships between specific manifestations of self-destructiveness and specific dimensions of emotional intelligence differ between women and men. One of the most important differences is the relationship between transgression (and risk) and ability to recognise emotions: it was positive in women and negative in men. What also seems puzzling is positive (although statistically non-significant) correlations between helplessness and emotional intelligence as a whole and specific abilities that make that up. Those correlations should be subject of further research.

Results of the research may prove useful in prophylactic and therapeutic work. The knowledge of the gender-differentiated relationships between specific categories of indirectly self-destructive behaviours and specific dimensions of emotional intelligence may allow to take prophylactic and therapeutic actions adjusted to the gender of individuals affected by those actions.
